# Effects of Strontium incorporation to Mg-Zn-Ca biodegradable bulk metallic glass investigated by molecular dynamics simulation and density functional theory calculation

**DOI:** 10.1038/s41598-020-58789-8

**Published:** 2020-02-13

**Authors:** Shih-Jye Sun, Shin-Pon Ju, Cheng-Chia Yang, Kai-Chi Chang, I-Jui Lee

**Affiliations:** 1 Department of Applied Physics, National University of Kaohsiung, Kaohsiung, 811 Taiwan; 2 Department of Mechanical and Electro-Mechanical Engineering, National Sun Yat-sen University, Kaohsiung, 804 Taiwan; 3 Department of Medicinal and Applied Chemistry, Kaohsiung Medical University, Kaohsiung, 807 Taiwan

**Keywords:** Structure of solids and liquids, Glasses

## Abstract

Molecular dynamics (MD) simulation and density functional theory (DFT) calculations were used to predict the material properties and explore the improvement on the surface corrosion resistance for the Mg_66_Zn_30_Ca_3_Sr_1_ bulk metallic glass (BMG). The Mg_66_Zn_30_Ca_4_ BMG was also investigated to realize the influence of the addition of Sr element on the material behaviors of Mg_66_Zn_30_Ca_4_. The Mg-Zn-Ca-Sr parameters of the next nearest-neighbor modified embedded atom method (2NN MEAM) potential were first determined by the guaranteed convergence particle swarm optimization (GCPSO) method based on the reference data from the density functional theory (DFT) calculation. Besides, using the 2NN MEAM parameters of the Mg-Zn-Ca-Sr system, the structures of Mg_66_Zn_30_Ca_4_ and Mg_66_Zn_30_Ca_3_Sr_1_ were predicted by the simulated-annealing basin-hopping (SABH) method. The local atomic arrangements of the predicted BMG structures are almost the same as those measured in some related experiments from a comparison with the calculated and experimental X-ray diffraction (XRD) profiles. Furthermore, the HA index analysis shows that the fractions of icosahedra-like local structures are about 72.20% and 72.73% for Mg_66_Zn_30_Ca_4_ and Mg_66_Zn_30_Ca_3_Sr_1_, respectively, indicating that these two BMG structures are entirely amorphous. The uniaxial tensile MD simulation was conducted to obtain the stress-strain relationship as well as the related mechanical properties of Mg_66_Zn_30_Ca_4_ and Mg_66_Zn_30_Ca_3_Sr_1_. Consequently, the predicted Young’s moduli of both BMGs are about 46.4 GPa, which are very close to the experimental values of 48.8 ± 0.2 and 49.1 ± 0.1 GPa for Mg_66_Zn_30_Ca_4_ and Mg_66_Zn_30_Ca_3_Sr_1_, respectively. However, the predicted strengths of Mg_66_Zn_30_Ca_4_ and Mg_66_Zn_30_Ca_3_Sr_1_ are about 850 and 900 MPa, both are slightly higher than the measured experimental values about 747 ± 22 and 848 ± 21 MPa for Mg_66_Zn_30_Ca_4_ and Mg_66_Zn_30_Ca_3_Sr_1_. Regarding the thermal properties, the predicted melting temperature of Mg_66_Zn_30_Ca_3_Sr_1_ by the square displacement (SD) profile is about 620 K, which is very close to the experimental melting temperature of about 613 K. The self-diffusion coefficients of Mg, Zn, Ca, and Sr elements were also calculated for temperatures near their melting points by means of the Einstein equation. The methodology can determine the diffusion barriers for different elements by utilizing these diffusion coefficients resulting in a fact that the diffusion barriers of Ca and Sr elements of Mg_66_Zn_30_Ca_3_Sr_1_ are relatively high. For the electronic properties predicted by the DFT calculation, the projected density of states (PDOS) profiles of surface Mg, Zn, Ca, and Sr elements clearly show that the addition of Sr into Mg_66_Zn_30_Ca_4_ effectively reduces the s and p orbital states of surface Mg and Zn elements near the Fermi level, particularly the p orbits, which suppresses the electron transfer as well as increases the surface corrosion resistance of Mg_66_Zn_30_Ca_4_. Consequently, this study has provided excellent 2NN MEAM parameters for the Mg, Zn, Ca, and Sr system by the GCPSO method to predict real BMG structures as well as by means of the DFT calculation to explore the electronic properties. Eventually, through our developed numerical processes the material properties of BMGs with different compositions can be predicted accurately for the new BMG design.

## Introduction

Metal possessing high strength, outstanding ductility, and high resistance to fracture has been considered as promising biomaterials for long-term implants^1–3^. The corrosion and wear of metallic implants could induce the biocompatibility problem, resulting in inflammation, cell apoptosis, and other destructive tissue reactions^4,5^. In that context, the salable products of Mg-based biodegradable implants have appeared in the market^6–9^. Alloys or BMG materials are one of approaches to improve the ductility and various mechanical properties and to enhance the corrosion resistance of metal implants. For understanding the BMG degradation behavior in different aqueous environments, several parameters including alloying element types, compositional fractions, impurities, and manufacturing method should be carefully considered because these factors have significantly influences on the secondary phase, microstructure, and surface structure of BMGs^10–15^. Furthermore, BMG shows complex behavior in the physiological environment when the dissolved oxygen, proteins, amino acids, chloride, and hydroxide ions are in proximity to BMG surface^16,17^.

Since magnesium alloys have their excellent biocompatibility, high biodegradability, and high mechanical strength while being lightweight^18–23^. Compared with other materials used in medical applications, magnesium alloys have advantages in short-term implantation, such as bone screws, bone plates, intramedullary nails, and temporary vascular stents to reduce patient discomfort. However, one main problem is that weak corrosion resistance in clinical reaction leads to its degradation in a short time^24,25^, resulting in the accumulation of hydrogen molecules around the implant, a delay in healing, and possible tissue necrosis. A more severe problem is the rapid decline in implant strength, leading to the loss of implant function before wound healing^26,27^. Therefore, improving the corrosion resistance of magnesium alloys is critical to expand their applications.

A practical method for improving magnesium alloy corrosion resistance is through surface coatings, such as calcium-deficient hydroxyapatite coatings^28^, CaP/chitosan/carbon nanotube coatings^29^, fluoride coatings^30^ and polycaprolactone fiber coating^31^, which forms a corrosion-resistant layer to protect the bulk of the magnesium alloy. Besides, surface treatments, an alternative is to change the magnesium alloy from crystalline to amorphous structure; that is, as magnesium-based metallic glasses (MGs)^32,33^. The atom arrangements of MGs having a homogeneous single-solid-solution phase are in the equilibrium state, which exceeds the solubility limit of the alloy element^31^. When a sufficient number of corrosion-resistant elements exist, the uniform passivation film exists due to the homogeneous single-phase property^34–36^, resulting in that the metallic glass exhibits higher corrosion resistance. For Mg_65_Ni_20_Nd_15_ and Mg_65_Cu_25_Y_10_ in strongly alkaline hydroxide electrolyte as discussed in Yao’s study^37^, the corrosion resistance content of these two BMGs is significantly improved compared to pure Mg, Mg_82_Ni_18_, and Mg_79_Cu_21_. The excellent corrosion resistance of magnesium-based MG can considerably reduce their degradation rate and inhibit hydrogen evolution, which prevents a rapid fall in strength and prolongs the biodegradation life.

As an alloy material composed of non-toxic elements, magnesium-containing metallic glasses have been widely observed for their biological applications^32,38,39^. Such as, Zberg *et al*.^40^ have noted that Mg_60+x_Zn_35-x_Ca_5_ MG with zinc content above 28% significantly inhibits the hydrogen precipitation within the implant due to the existence of the passivated ZnO/ZnCO_3_ layer. Besides, clinical trials have shown that Mg-Zn-Ca MGs have excellent biocompatibility, and almost no cavity around the implant was found even though a large amount of hydrogen appear^40^; Gu found the metallic glass with the Mg-Zn-Ca content displays better biocompatibility than that of pure magnesium material during the uniform corrosion process^32^. In Chan’s study^41^, the cell adhesion extent of gelatin surface coated by Mg_67_Zn_28_Ca_5_ was significantly improved.

Strontium (Sr), a bone-seeking element, is in the same column of periodic table as calcium and its chemical and biological properties are very similar to those of calcium^39^. It has been reported that Sr can stimulate the replication of bone cells and protein synthesis and inhibit bone resorption after the bone mass increases and the bond strength is improved^38,39^. Therefore, Sr could be a potential element for addition to MgZnCa BMG to improve its mechanical and electronic properties. In Li’s study^42^, the experimental results showed that MgZnCa BMG incorporating Sr at a small Sr fraction (<1.5%), the mechanical properties and corrosion resistance of MgZnCa BMG are significantly improved, which promote its potential as a promising biodegradable material. Li’s study also indicated Mg, Ca, and Sr play an irreplaceable role in the bone formation. Therefore, to understand whether the mechanical properties and the biocompatibility of pure Zn can be further improved, three ternary alloys (ZnMgCa, ZnMgSr, and ZnCaSr) were fabricated. The experimental measures demonstrated the mechanical properties of pure Zn can be significantly improved after alloyed with Mg, Ca, or Sr. Besides, i*n vitro*, according to the hemolytic rate test and cell viability test results, the hemocompatibility and cytocompatibility of MgZnCaSr BMGs are considerably enhanced compared to those of pure Zn^43^.

Since it is still a challenge to study the detailed local atomic arrangement around each component element of BMG directly from the experimental methods, instead, theoretical simulation used various computational models for predicting the mechanical, thermal and other crucial properties of biodegrading BMG implants^44–46^. Such as molecular dynamics (MD) simulation, which has been successfully in studying the structural, mechanical, and thermodynamic properties of MgZnCaSr BMG was used for MgCa BMGs in our previous study^47^. Similarly, the density functional theory (DFT) calculation was also used to explore the electronic properties of MgZnCaSr BMG as well as understand the mechanism of the improved corrosion resistance of the Sr-incorporated MgZnCa BMG. In reality, there are so many complexities in the environments of BMG implant as aforementioned it is too complicated to have overall considerations in the theoretical work. However, a proper simulation based on proper models and parameters still could well describe many properties of BMG comparable to experimental results.

## Simulation Model

In order to simulate the Mg-Zn-Ca-Sr alloy system by molecular simulation, the second nearest neighbor modified embedded atomic method (2NN MEAM)^48,49^ was used to describe the interaction between different atomic pairs using the potential function.

The 2NN MEAM potential has the following form:

1 $$E=\sum _{i}[{F}_{i}(\overline{{\rho }_{i}})+\frac{1}{2}\sum _{j(\ne i)}{\phi }_{ij}({R}_{ij})]$$

where F is the embedding energy, which is a function of the atomic electron density $\overline{{\rho }_{i}}$, and ${\phi }_{ij}$ is the pair interaction potential. The atomic electron density $\overline{{\rho }_{i}}$ comes from the combination of four complicated formulas, which consider the influence of electrons in s, p, d, and f orbitals, respectively. The detailed introduction of 2NN MEAM can be seen in the corresponding original studies^48,49^ and is not introduced here. The guaranteed convergence particle swarm optimization (GCPSO)^50^ was used to determine the 2NN MEAM parameters for the Mg-Zn-Ca-Sr system. The GCPSO is a variable optimization process based on an objective function. It is the sum of the squared differences between material properties obtained by the potential function and those obtained by density functional theory (DFT). The detailed 2NN MEAM parameter fitting process and the fitted parameters for the Mg-Zn-Ca-Sr system can be seen in the supplementary information files including a PDF file for introducing the detailed parametrization process, MEAM potential file for single element parameters (MgZnCaSr.meam), and MEAM potential file for the cross-element parameters (MgZnCaSr_cross_element.meam).

After the 2NN MEAM parametrization process, the simulated-annealing basin-hopping (SABH)^51^ method was used to generate the stable amorphous BMG structure along the search direction of the local minimum structure with the higher energy. Because Mg_66_Zn_30_Ca_3_Sr_1_ has been identified as possessing the best corrosion resistance ability by Li^42^, this BMG composition was used in this study. To investigate the difference of BMG structural and material properties after the Sr incorporation, Mg_66_Zn_30_Ca_4_ was also considered.

**Figure 1:**
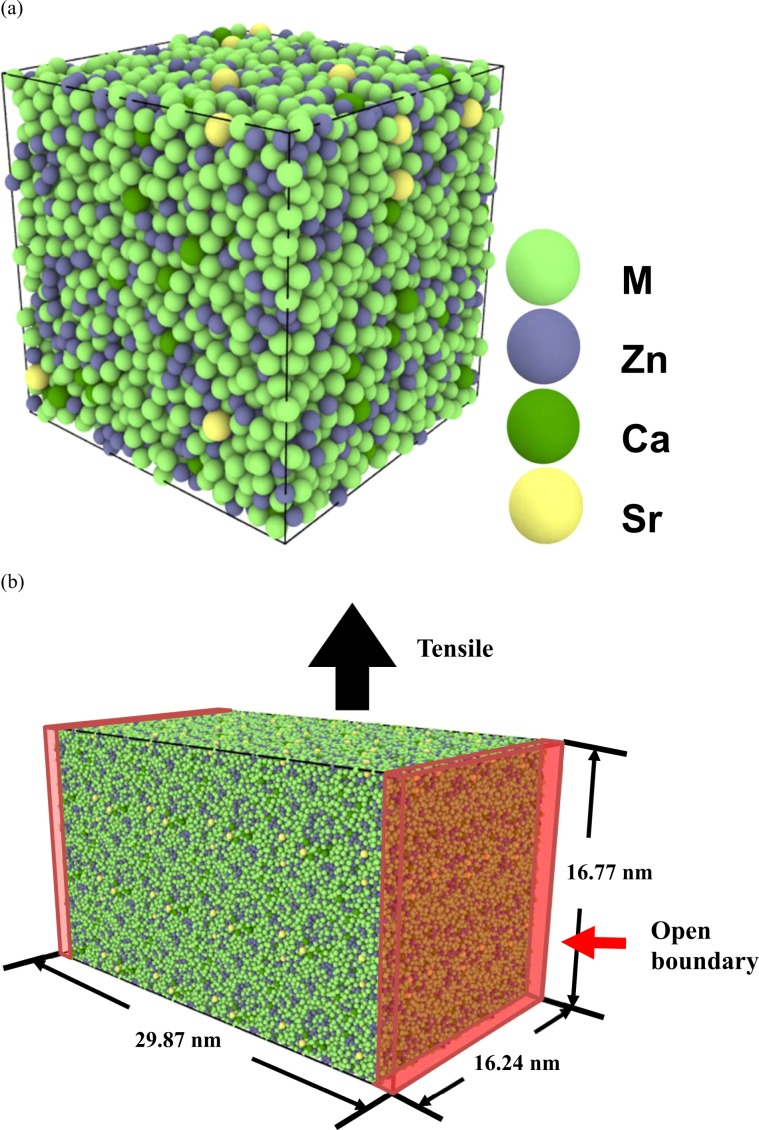
(**a**) The unit cell of Mg_66_Zn_30_Ca_3_Sr_1_ used for simulating by the SABH method; (**b**) The 3 × 2 × 2 supercell for the tensile simulation.

The unit cell with 8000 atoms was used for both BMGs, and a schematic diagram of the Mg_66_Zn_30_Ca_3_Sr_1_ unit cell is displayed in Fig. 1(a). The 3 × 2 × 2 supercell with 96,000 atoms for the MD uniaxial tension simulation is illustrated in Fig. 1(b). One open boundary condition was applied to modeling the BMG surface, and the periodic boundary conditions were used for the other two boundaries. All SABH processes and MD simulations were conducted by LAMMPS, a large-scale atomic/molecular parallel simulator developed by Plimpton^52^. The strain rate of 5 × 10^8^ m·s^−1^ was used, below which the stress-strain profiles are very close. The detailed information for the MD tensile simulation and the stress calculation can be also seen in the supplementary file.

The DMol3 package was used for all DFT calculations; the generalized gradient approximation (GGA) with the parameterization of PBEsol was used, and the energy tolerance y in the self-consistent field calculations was 2.72 × 10–5 eV. Regarding the precision of the calculation, the orbital cutoff quality and k mesh points were set to medium and 4 × 4 × 4, respectively.

## Results and Discussion

**Figure 2:**
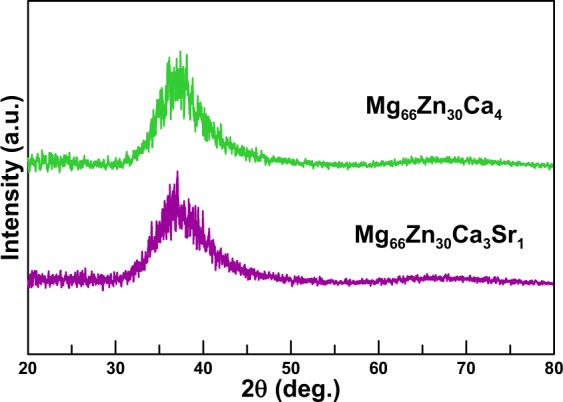
The XRD profiles of Mg_66_Zn_30_Ca_4_ and Mg_66_Zn_30_Ca_3_Sr_1_.

The unit cells of Mg_66_Zn_30_Ca_3_Sr_1_ and Mg_66_Zn_30_Ca_4_ were first characterized by the X-ray diffraction (XRD) calculation implemented by LAMMPS. Figure 2 shows the simulated XRD profiles of Mg_66_Zn_30_Ca_4_ and Mg_66_Zn_30_Ca_3_Sr_1_, respectively. No distinct crystallization peak can be seen in these two XRD profiles, instead, broad peaks between 30°–47° can be seen. These simulated Mg_66_Zn_30_Ca_4_ and Mg_66_Zn_30_Ca_3_Sr_1_ XRD profiles are almost identical to the corresponding experimental XRD profiles of Mg_66_Zn_30_Ca_4_^40^ and Mg_66_Zn_30_Ca_3_Sr_1_^53,54^, which indicates that the SABH method with the fitted 2NN MEAM parameters can obtain the Mg_66_Zn_30_Ca_4_ and Mg_66_Zn_30_Ca_3_Sr_1_ structures as shown from the experiment.

**Figure 3:**
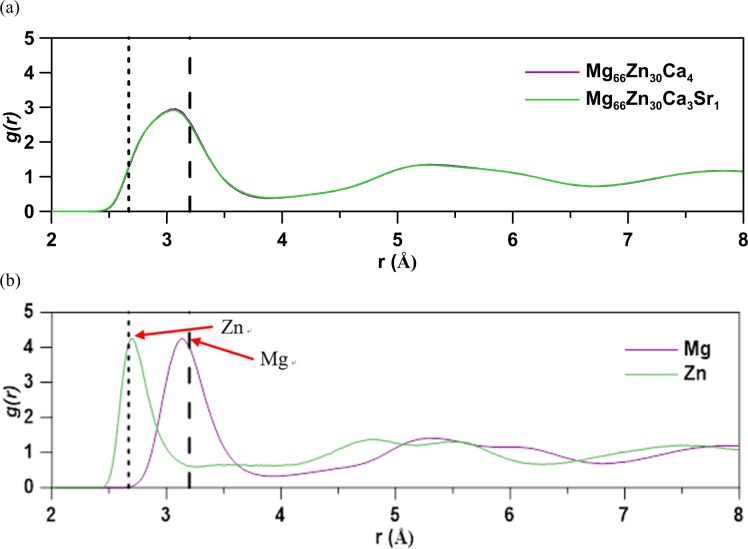
(**a**) The radial distribution function (RDF) profiles for Mg_66_Zn_30_Ca_4_ and Mg_66_Zn_30_Ca_3_Sr_1_; (**b**) the partial radial distribution function (PRDF) profile for Mg_66_Zn_30_Ca_3_Sr_1_. The first peaks of HCP Mg and Zn are indicated with vertical dashed lines for comparison.

Figure 3(a) shows the radial distribution function (RDF) profiles of Mg_66_Zn_30_Ca_4_ and Mg_66_Zn_30_Ca_3_Sr_1_. One can see that these two RDF profiles match closely to each other, which represents that the minor Sr-incorporated MgZnCa BMG does not change the local atomic arrangement of MgZnCa BMG. The first RDF peaks range from 2.5 to 4.0 Å, and the second peaks ranging between 4.0 and 6.8 Å become wider, which reveals a fact that the Mg_66_Zn_30_Ca_4_ and Mg_66_Zn_30_Ca_3_Sr_1_ are amorphous and only display short-range order. Figure 3(b) shows the partial radial distribution function (PRDF) profiles of Mg-Mg and Zn-Zn pairs in Mg_66_Zn_30_Ca_3_Sr_1_. Because both PRDF profiles very similar, only the PRDF profiles for Mg_66_Zn_30_Ca_3_Sr_1_ are displayed in Fig. 3(b). In comparison with the HCP Mg and Zn RDF profiles, the first peaks of HCP Mg and Zn are also represented by the vertical dashed lines in Fig. 3(b). One can see that only the distinct first PRDF peaks exist for both Mg and Zn, the highest two-element fraction, which also confirms that only short-range order exists within the Mg_66_Zn_30_Ca_3_Sr_1_. The first peaks of Mg-Mg and Zn-Zn pairs of Mg_66_Zn_30_Ca_3_Sr_1_ appear at about 3.16 and 2.70 Å, respectively, while the experimentally measured first RDF peaks of HCP Mg and HCP Zn are at about 3.20 and 2.67 Å^55,56^. Thus, distances of Mg-Mg and Zn-Zn in Mg_66_Zn_30_Ca_3_Sr_1_ are close to those of HCP Mg and HCP Zn.

**Figure 4:**
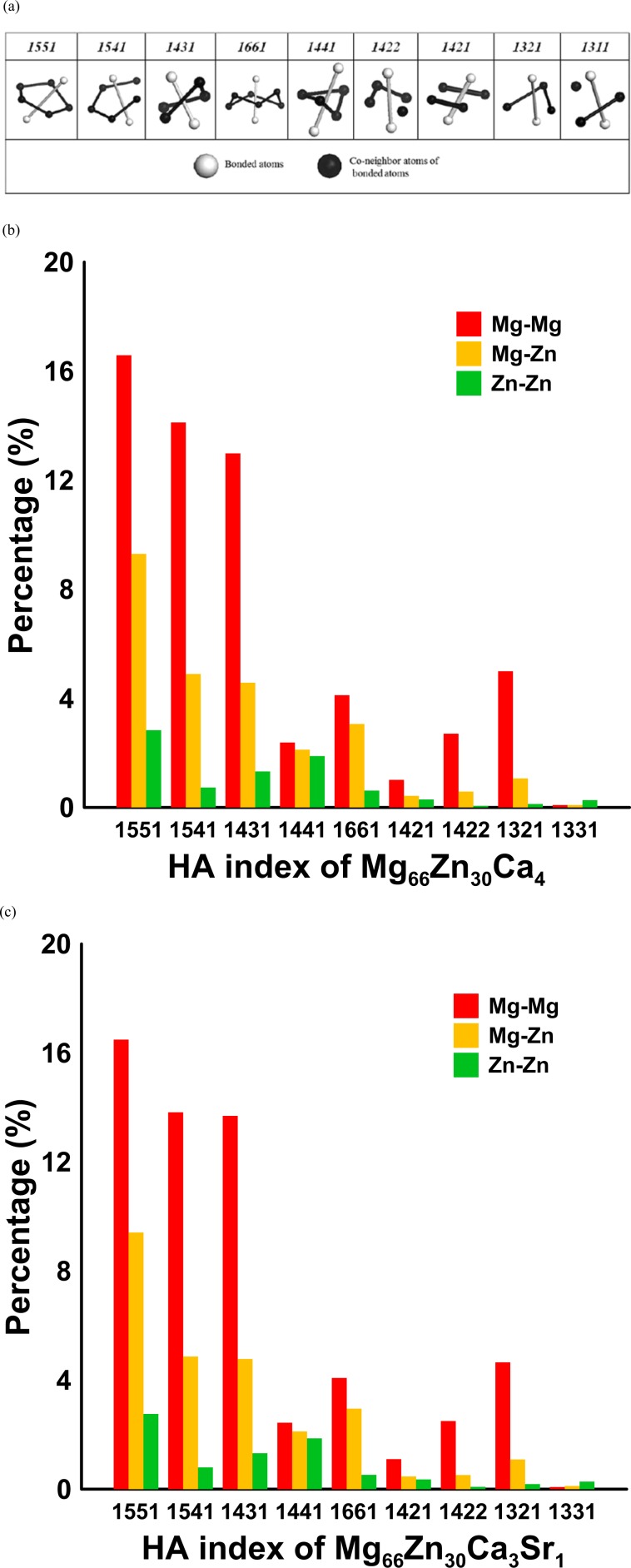
(**a**) Schematic diagrams corresponding to several characteristic HA indexes; HA indexes for different pairs in (**b**) Mg_66_Zn_30_Ca_4_ and (**c**) Mg_66_Zn_30_Ca_3_Sr_1_.

The local atomic arrangements of Mg_66_Zn_30_Ca_4_ and Mg_66_Zn_30_Ca_3_Sr_1_were further studied by means of the HA pair analysis method. The detailed definition of the HA index can be found elsewhere^57^. The schematic diagrams of all HA indexes are presented in Fig. 4(a). The HA indexes of 1421 and 1422 are responsible for F.C.C. and H.C.P. crystal structures, and 1431, 1541, and 1551, which have the highest fraction occupation in the amorphous or liquid state are used to identify the local icosahedral structures. The HA indices 1661 and 1441 are used to identify local B.C.C. structures. The 1321 and 1311 are the stacking types related to rhombohedral pairs, which can be considered as by-products accompanying with the accumulation of icosahedron atoms (1551 type).

Because the total occupation of Mg and Zn atoms in Mg_66_Zn_30_Ca_4_ and Mg_66_Zn_30_Ca_3_Sr_1_ is 96% and the atomic radius of Mg is larger than that of Zn by 17.04%, by HA analyses only Mg-Mg, Mg-Zn, and Zn-Zn pairs were considered. Figure 4(b,c) show the Mg-Mg, Mg-Zn, and Zn-Zn HA index distributions for Mg_66_Zn_30_Ca_4_ and Mg_66_Zn_30_Ca_3_Sr_1_. These two HA distributions are very similar, indicating the local atomic arrangements of both BMGs are very similar and consistent with the RDF profiles as shown in Fig. 3(a). The fraction of icosahedral-like local structures (1551, 1541, and 1431) of Mg_66_Zn_30_Ca_4_ and Mg_66_Zn_30_Ca_3_Sr_1_ are about 72.20% and 72.73%, respectively. The high icosahedron fraction indicates that the amorphous icosahedral-like local structures dominate Mg_66_Zn_30_Ca_4_ and Mg_66_Zn_30_Ca_3_Sr_1_. In particular, among all icosahedral-like local structures, the perfect icosahedrons (1551) have the highest fractions of Mg-Mg, Mg-Zn, and Zn-Zn pairs.

Table 1 shows the average coordination numbers (CNs) for different atoms in Mg_66_Zn_30_Ca_4_ and Mg_66_Zn_30_Ca_3_Sr_1_ as well as the partial coordination number of different atomic pairs. The CNs were calculated by determining the number of the nearest neighbor atoms around the reference atom. The cutoff length for each CN calculation is estimated from the first minimum distance of the corresponding RDF profile. The average distances of the nearest neighbor atoms in different pairs are also listed in the corresponding parentheses. For the reference Mg, Zn, and C atoms in Mg_66_Zn_30_Ca_4_ and Mg_66_Zn_30_Ca_3_Sr_1_, the CNs of different pairs, total CN, and the average distances are almost the same, which indicates the microstructures of both BMGs are almost identical. For the reference, Sr atom, the total CN of Sr is the largest because the atomic size of Sr is the largest among these four elements presented in Mg_66_Zn_30_Ca_3_Sr_1_ resulting in the space around this atom containing more nearest neighbor atoms.

**Table 1:** Average coordination numbers (CNs) of Mg, Zn, Ca, and Sr atoms in Mg_66_Zn_30_Ca_4_ and Mg_66_Zn_30_Ca_3_Sr_1_. The values in the parentheses are the average distances of the nearest neighbor atoms. The first and the second atoms in each pair type are the reference atom and the nearest neighbor atom, respectively.

Type N_ij_(D_ij_)	Mg_66_Zn_30_Ca_4_	Mg_66_Zn_30_Ca_3_Sr_1_
Mg-Mg	8.83(3.26)	8.82(3.27)
Mg-Zn	3.81(3.06)	3.79(3.05)
Mg-Ca	0.62(3.46)	0.45(3.46)
Mg-Sr	n/a	0.15(3.62)
Mg Total	13.26	13.22
Zn-Mg	8.38(3.06)	8.34(3.05)
Zn-Zn	3.47(3.16)	3.37(3.16)
Zn-Ca	0.62(3.34)	0.47(3.34)
Zn-Sr	n/a	0.17(3.55)
Zn Total	12.47	12.35
Ca-Mg	10.22(3.46)	9.91(3.46)
Ca-Zn	4.62(3.34)	4.73(3.34)
Ca-Ca	0.45(3.79)	0.47(3.81)
Ca-Sr	n/a	0.11(3.90)
Ca Total	15.28	15.21
Sr-Mg	n/a	10.04(3.62)
Sr-Zn	n/a	5.16(3.55)
Sr-Ca	n/a	0.33(3.90)
Sr-Sr	n/a	0.10(2.85)
Sr Total	n/a	15.63

Warren-Cowley chemical short-sequence (CSRO) analysis^58^ was used to evaluate the attraction and repulsion between each pair in Mg_66_Zn_30_Ca_4_ and Mg_66_Zn_30_Ca_3_Sr_1_. The CSRO parameters evaluated the affinity of a reference atom relative to its nearest neighbor atoms according to its CN information shown in Table 1. The CSRO parameter of the *i*th atom relative to the *j*th $({\alpha }_{ij})$ atom for an individual pair type is defined as:

2 $${\alpha }_{ij}=1-\frac{{N}_{ij}}{{c}_{j}{N}_{i}}$$

where *N*_*ij*_ represents the partial CN for the *i*th atom relative to *j*th atom shown in Table 1, and *c*_*j*_ and *N*_*i*_ are the fractions of the *j*th atom within the alloy and the average CN of the *i*th atom, respectively. The value of *c*_*j*_ by *N*_*i*_ is an ideal partial CN for the *i*th atom relative to the nearest neighbor *j*th atom if all element atoms are completely uniformly distributed. The CSRO parameters of all pair types in Mg_66_Zn_30_Ca_4_ and Mg_66_Zn_30_Ca_3_Sr_1_ are listed in Table 2. The CSRO values for Mg_66_Zn_30_Ca_4_ and Mg_66_Zn_30_Ca_3_Sr_1_ are very similar to the values of the corresponding reference Mg and Zn pairs. The values for the CSRO of Mg-Mg pairs of both BMGs are relatively smaller when compared to other pairs, which represents the most abundant Mg element in both BMGs serves as the ideal solution medium for the mixture of other elements. The CSRO values of Mg-Ca and Zn-Ca pairs are relatively lower (negative), indicating that both Mg and Zn elements have a higher affinity relative to the Ca atom. For Mg_66_Zn_30_Ca_3_Sr_1_, the CSRO values of Mg-Sr and Zn-Sr are negative, and the CSRO values of Ca-Sr and Sr-Sr are positive. It indicates Mg and Zn elements, the first two highest fractions in Mg_66_Zn_30_Ca_3_Sr_1_, have a strong affinity relative to Sr. Thus, the addition of Sr would likely increase the glass-forming ability of MgZnCa BMG, as found in the experimental study^42^. It is supposed that the increase in the glass-forming ability of MgZnCa BMG is owing to a relatively large atomic size of the Sr atom, which extends the space and increases the nearest neighbor atoms.

**Table 2:** CSRO parameters (*α*_*ij*_) for all pair type of Mg_66_Zn_30_Ca_4_ and Mg_66_Zn_30_Ca_3_Sr_1_.

Type *α*_*ij*_	Mg_66_Zn_30_Ca_4_	Mg_66_Zn_30_Ca_3_Sr_1_
Mg-Mg	−0.0090	−0.0114
Mg-Zn	0.0420	0.0436
Mg-Ca	−0.1673	−0.1361
Mg-Sr	n/a	−0.1504
Zn-Mg	−0.0187	−0.0234
Zn-Zn	0.0722	0.0921
Zn-Ca	−0.2338	−0.2749
Zn-Sr	n/a	−0.3929
Ca-Mg	−0.0129	0.0127
Ca-Zn	−0.0068	−0.0353
Ca-Ca	0.2638	−0.0226
Ca-Sr	n/a	0.2879
Sr-Mg	n/a	0.0267
Sr-Zn	n/a	−0.1013
Sr-Ca	n/a	0.3067
Sr-Sr	n/a	0.3600

The first and the second atoms in each pair are the reference atom and the nearest neighbor atom, respectively.

**Figure 5:**
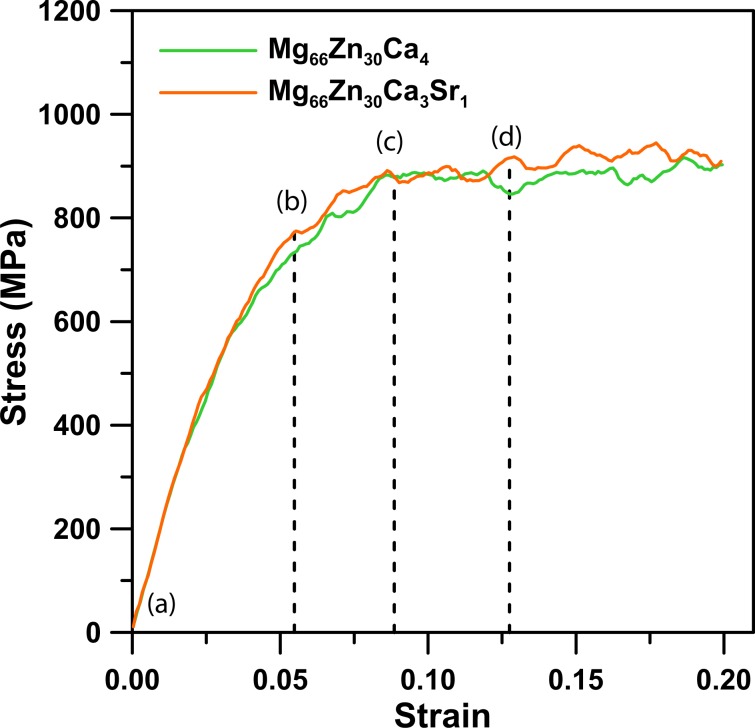
The stress-strain curves of Mg_66_Zn_30_Ca_4_ and Mg_66_Zn_30_Ca_3_Sr_1_.

The uniaxial tensile MD simulation at 5 K was carried out to obtain the mechanical properties of Mg_66_Zn_30_Ca_4_ and Mg_66_Zn_30_Ca_3_Sr_1_. Figure 5 displays the stress-strain profiles of both materials under the tension simulation. One can see that the stress of both BMGs increases linearly with the increase of strain from 0 to about 0.062, which reveals a fact that the elastic behaviors of both materials are within this strain range. The Young’s modulus obtained from the slopes of stress-strain curves of both materials within the range of 0 ~ 0.02 both is about 46.4 GPa, which are slightly lower than the experimental values of 48.8 ± 0.2 and 49.1 ± 0.1 GPa for Mg_66_Zn_30_Ca_4_ and Mg_66_Zn_30_Ca_3_Sr_1_^42^ by 4.9% and 5.5%, respectively. The predicted strengths of Mg_66_Zn_30_Ca_4_ and Mg_66_Zn_30_Ca_3_Sr_1_ are about 850 MPa and 900 MPa, and both values are slightly higher than the experimentally measured values about 747 ± 22 and 848 ± 21 MPa, respectively. Furthermore, within the strain range between 0.062~0.089, the stresses of Mg_66_Zn_30_Ca_4_ and Mg_66_Zn_30_Ca_3_Sr_1_ show a slowing growth with an increase of strain. When the strain exceeds 0.089, the stresses of both BMGs fluctuate and eventually reach maximum values of about 850 and 900 MPa, respectively. As aforementioned in the introduction, the purpose of the study is to find an improvement of the corrosion resistance for the implant materials as well as increase the structural strength. The calculated stress-strain curves show that the addition of Sr to MgZnCa BMG can enhance its strength, resulting in the maximum stress of Sr-doped MgZnCa glass being slightly higher than that of undoped MgZnCa BMG. Obviously, the strength improvement is owing to a relatively large atomic size of Sr atom, which increases the atomic coordinate numbers as well as enhances the strength of stress.

**Figure 6:**
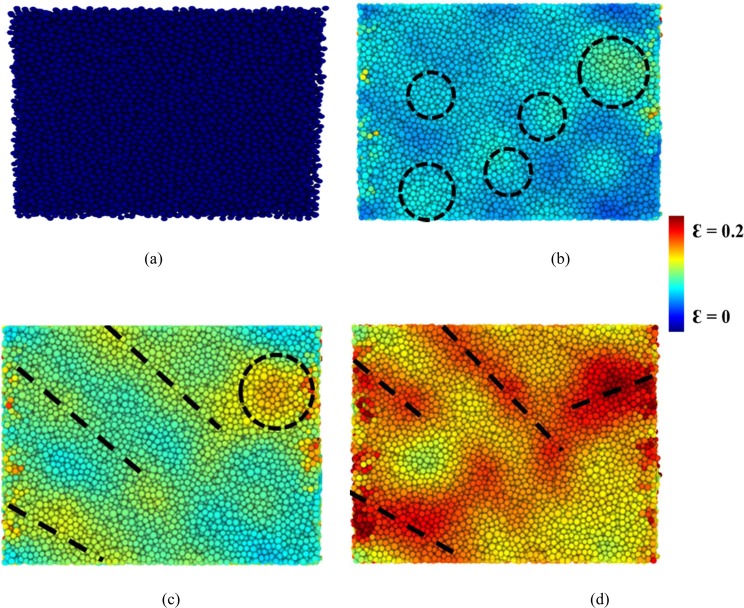
The shear band and local shear transition zone evolution within Mg66Zn30Ca3Sr1 at strains of (**a**) 0, (**b**) 0.062, (**c**) 0.089, and (**d**) 0.127.

The atomic local shear strain *η*_*i*_^Mises^ for an individual atom^59^ was used to determine the evolution of the shear transformation zones (STZ) and the formation of shear bands within Mg_66_Zn_30_Ca_4_ and Mg_66_Zn_30_Ca_3_Sr_1_ during the tensile variations. In fact, All the atomic *η*_*i*_^Mises^ values were calculated directly by OVITO^60^ and the structure is at free strain initially, in which the *η*_*i*_^Mises^ values of all atoms are 0. Figure 6(a–d) shows snapshots of Mg_66_Zn_30_Ca_3_Sr_1_ with atomic η_i_^Mises^ values at strains of 0, 0.062, 0.089, and 0.127, labeled respectively as (a)-(d), corresponding to the stress-strain curves in Fig. 5. Owing to the STZ evolution of Mg_66_Zn_30_Ca_4_ is very similar to that of Mg_66_Zn_30_Ca_3_Sr_1_, only the STZ process of Mg_66_Zn_30_Ca_3_Sr_1_ is discussed. Figure 6(a) shows the snapshot of a reference structure at a free strain. As indicating by the dashed circles in Fig. 6(b), the initial stages of STZs occur at the strain 0.062 and distribute randomly within the Mg_66_Zn_30_Ca_3_Sr_1_. Feng^61^ has concluded that the random distribution of STZs is primarily due to the structural heterogeneity of BMGs at the initial state. At the strain 0.089, as indicating in Fig. 6(c) by black dashed lines, an extension of these STZs commence forming several shear bands. Actually, even more, shear bands occur at a strain of 0.127, which is quite evident in Fig. 6(d).

**Figure 7:**
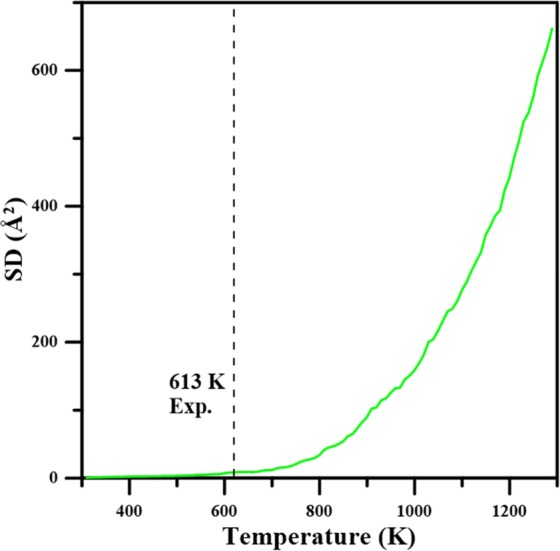
Average system square displacement (SD) as a function of temperature for Mg_66_Zn_30_Ca_3_Sr_1_ during the heating process.

The thermal behaviors of Mg_66_Zn_30_Ca_3_Sr_1_ were investigated by the MD temperature elevation process, which began at an initial temperature of 300 K and rose to 1300 K. This process utilized the TtN method^62^, which combines the Parrinello-Rahman variable shape size ensemble with the Nosé–Hoover thermostat. The model for the temperature elevation process is the same as presented in Fig. 1(a). The TtN method was used to maintain the temperature at a constant under free stress. The heating process processed in the increasing temperature by 10 K increments and each increment was accompanied by a relaxation process in 10 ps before the subsequent temperature increases. The square displacement (SD) profile during the temperature elevation was used to observe the melting behavior of Mg_66_Zn_30_Ca_3_Sr_1_. The definition of SD at time t is shown in Eq. (3):

3 $${\rm{SD}}({\rm{t}})=\frac{{\sum }_{i}^{N}{[{r}_{i}(t)-{r}_{i}(0)]}^{2}}{N}\,$$

where ${r}_{j}(0)$ is the position of the *i*th atom at time 0, ${r}_{j}(t)$ represents the position of the *i*th atom at time *t*, and N is the total atom number of Mg_66_Zn_30_Ca_3_Sr_1_. It should be noted that the system temperature increases with the simulation time which corresponds to the temperature of the system. Figure 7 shows the SD variation during the heating process for Mg_66_Zn_30_Ca_3_Sr_1_, in which the experimental melting temperature (T_m_) of Mg_66_Zn_30_Ca_3_Sr_1_ is about 613 K^42^, indicated by the dashed line. The SD is linearly proportional to the temperature increase from 300 K to about 620 K, while the SD profile shows a parabolically increasing growth when the temperature exceeds 620 K. Expectedly, within the SD linear range (300–620 K), all atoms undergo thermal vibrations and fluctuate around their equilibrium positions. Instead, when the temperature exceeds 620 K, the kinetic energies of atoms overcome the bonding energies of their equilibrium states and the local structures change significantly. Consequently, 620 K can be regarded as the T_m_ of Mg_66_Zn_30_Ca_3_Sr_1_, which is close to the experimental value, about 613 K. This reveals a fact that the thermal behavior of Mg_66_Zn_30_Ca_3_Sr_1_ can be reflected in the fitted 2NN MEAM parameters through the PSO algorithm.

**Figure 8:**
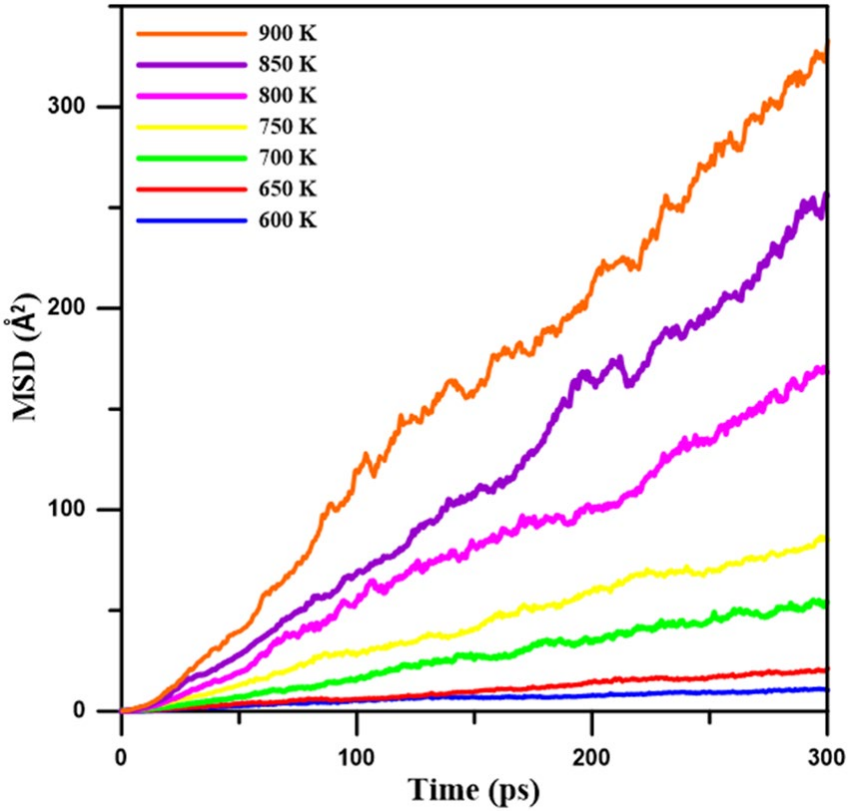
Mean-square displacement profiles (MSD) for Mg_66_Zn_30_Ca_3_Sr_1_ at different temperatures close to the melting point in the range from about 600 K to 900 K.

The mean-square displacement (MSD) profiles at temperatures near the T_m_ of Mg_66_Zn_30_Ca_3_Sr_1_ were utilized to investigate the diffusion behaviors of Mg, Zn, Ca, and Sr atoms. The MSD is defined as,

4 $${\rm{MSD}}=\frac{\langle {\sum }_{{\boldsymbol{i}}}^{{\boldsymbol{N}}}{[{{\boldsymbol{r}}}_{{\boldsymbol{i}}}({\boldsymbol{t}})-{{\boldsymbol{r}}}_{{\boldsymbol{i}}}({t}_{0})]}^{2}\rangle }{{\boldsymbol{N}}}\,$$

where *r*_*i*_*(t)* is the position of the *i*th atom at delay time *t*, and *r*_*i*_*(t*_0_) means the position of the corresponding atom at reference time *t*_0_; *N* is the total atom number. Figure 8 displays MSD profiles of Mg_66_Zn_30_Ca_3_Sr_1_ at 600, 650, 700, 750, 800, 850, and 900 K, it is clear that the slopes of MSD profiles increase with an increase of temperature. It is well-known that MSD profiles are linear with the delay time over the long term, and thus the diffusion coefficients of Mg_66_Zn_30_Ca_3_Sr_1_ can be obtained from the slopes of MSD profiles after a longe delay time by the Einstein equation:

5 $${\rm{D}}=\frac{1}{6N}\mathop{\mathrm{lim}}\limits_{t\to \infty }\frac{d}{dt}{\rm{MSD}}$$

where *D* is the self-diffusion coefficient, and *N* is the number of atoms. The MSD profiles of different elements at different temperatures were obtained from the Einstein equation for the Mg, Zn, Ca, and Sr diffusion coefficients of Mg_66_Zn_30_Ca_3_Sr_1_ at different temperatures. The formula of the Arrhenius equation which describes the diffusion coefficient at different temperatures, D(T)^63^ is:

6 $${\rm{D}}({\rm{T}})={D}_{0}\times {e}^{(\frac{-{\rm{Q}}}{{\rm{RT}}})}$$

**Figure 9:**
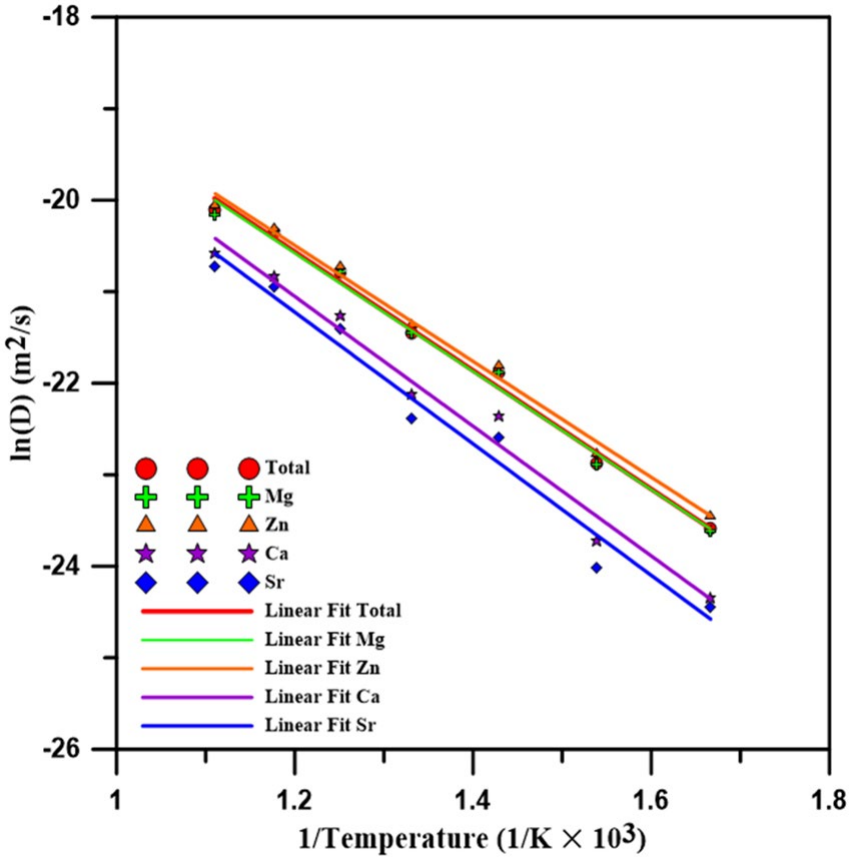
The diffusion coefficient of logarithm profiles as a function of the inverse of temperature for Mg_66_Zn_30_Ca_3_Sr_1_, Mg, Zn, Ca, and Sr, respectively.

In which Q is the activation energy, T is the temperature, D_0_ is the pre-exponential factor, and R is the Boltzmann constant. For the calculation of the diffusion barrier Q from the profiles of ln(D) versus 1/T for total, Mg, Zn, Ca, and Sr, the results are displayed in Fig. 9. Apparently, all ln(D) profiles decrease linearly with 1/T. Because the diffusion barriers of the total, Mg, Zn, Ca, and Sr atoms can be derived from the slopes of the ln(D) profiles, Table 3 lists the *D*_0_ and all diffusion barrier values. The diffusion barriers of the total, Mg, Zn, Ca, and Sr atoms are 53.77, 53.72, 52.63, 58.91, and 59.76 KJ mol^−1^, respectively. Obviously, the diffusion barriers of Ca and Sr atoms are relatively higher than those of Mg and Zn because the atomic size of Ca and Sr are relatively large and have more nearest neighbor atoms, as shown in Table 1. It is also expected that Mg and Zn atoms could diffuse easier with the increase of the temperature.

**Table 3:** The estimated pre-exponential factor (D_0_) and activation energy (Q) of Mg_66_Zn_30_Ca_3_Sr_1_.

Proportion	Type	*D*_0_(m^2^s^−1^)	*Q*(kJ mol^−1^)
Mg_66_Ca_30_Ca_3_Sr_1_	Total	2.80 × 10^−6^	53.77
	Mg	2.68 × 10^−6^	53.72
	Zn	2.50 × 10^−6^	52.63
	Ca	3.51 × 10^−6^	58.91
	Sr	3.36 × 10^−6^	59.76

DFT calculations were employed to investigate the electronic properties of Mg_66_Zn_30_Ca_4_ and Mg_66_Zn_30_Ca_3_Sr_1_ with the same DFT setting for the PSO force-matching process. Because DFT calculations take a long calculation time than that of 2NN MEAM potential calculations, only 400 atoms in a system were considered. In here, the SABH method was used to obtain the amorphous structures of Mg_66_Zn_30_Ca_4_ and Mg_66_Zn_30_Ca_3_Sr_1_. It is worth mentioning that the surfaces of Mg_66_Zn_30_Ca_4_ and Mg_66_Zn_30_Ca_3_Sr_1_ were constructed by expanding the simulation box length in the z dimension to investigate the surface electronic behaviors, and during the DFT geometrical optimization process, the atoms within 10 Å from the bottom surface were fixed while other atoms were set as movable.

**Figure 10:**
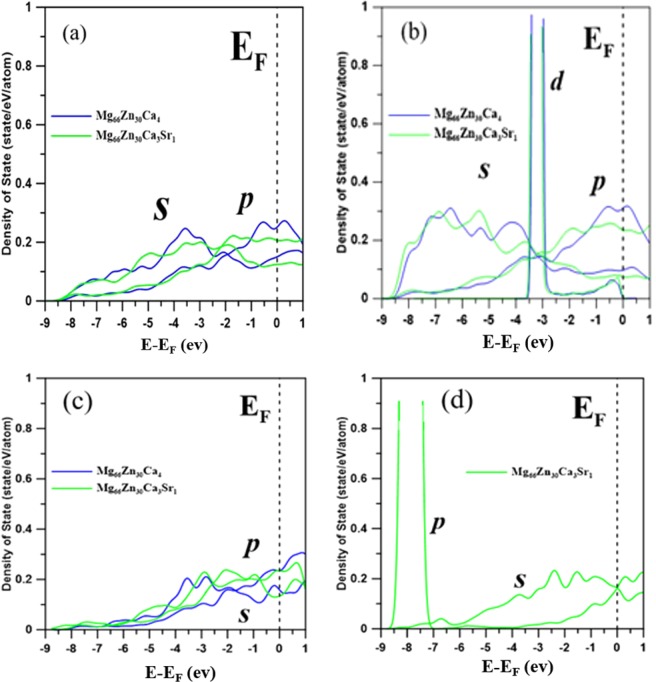
Projected density of states (PDOS) for (**a**) Mg, (**b**) Zn, and (**c**) Ca atoms on the surface of Mg66Zn30Ca4 and Mg_66_Zn_30_Ca_3_Sr_1_, and PDOS profile for (**d**) Sr atoms on the surface of Mg_66_Zn_30_Ca_3_Sr_1_ BMG. The energy is given relative to the Fermi level (E_F_).

Corrosion is an oxidation process that involves an intense charge transfer between the host and the oxidation driver. It is rational, reducing the charge transfer will suppress the corrosion. Because the valence electrons can proceed charge transfer better than the electrons in the inner orbitals, resulting in the oxidation process by electrons in the p orbital is more efficient than that by electrons in the s orbital. Figure 10 shows the projected density of states (PDOS) of s, p, and d orbitals for surface atoms of Mg_66_Zn_30_Ca_4_ and Mg_66_Zn_30_Ca_3_Sr_1_. Figure 10(a,b), show a fact that the addition of Sr to Mg_66_Zn_30_Ca_4_ causes the density of states of p orbital (p-PDOS) of surface Mg and Zn atoms near the Fermi-level to be lower than that of Mg_66_Zn_30_Ca_4_. The decrease of p-PDOS near the Fermi level implies the valence electrons in the p orbitals decreasing, which could, therefore, decrease the charge transfer from Mg and Zn atoms during the corrosion oxidation process. In other improvement words, the addition of Sr indeed enhances the corrosion resistance of MgZnCa glasses. It should be noted that the p-PDOS of surface Ca atoms rarely changes, as shown in Fig. 10(c), revealing the added Sr does not alter the electronic properties of Ca. In Fig. 10(d), the PDOS profiles of s and p orbitals of surface Sr atoms near the Fermi level are virtually the same, implying that the s and p orbitals of Sr strongly couple with the p orbitals of Mg and Zn. The improvement of the corrosion oxidation by the addition of Sr is owing to the increase of the nearest neighbor atoms (such as Mg and Zn) around Sr, which makes more electrons from the nearest neighbor atoms participate in bonding with Sr atoms. It leads to a suppression of the electrons involving the charge transfer during the oxidation process. It is worth mentioning that the energy of bound electrons move to states below the Fermi-level, which interprets a decrease of the p-PDOS of surface Mg and Zn atoms near the Fermi-level in Mg_66_Zn_30_Ca_3_Sr_1_.

Expectedly, our simulation methods conduct us to design new BMG materials processing strong mechanical structure and high corrosion resistance. Based on our results we propose that adds the relatively larger atomic size of atom to replace Ca concentration will improve both properties, such as Sr or Ba, and we believe the latter one will be much better than the former. Since the simulation of BMG Mg_66_Zn_30_Ca_3_Ba_1_ is our work in the future.

## Conclusion

The molecular dynamics simulation has been performed to investigate the structural, mechanical, and thermal properties of Mg_66_Zn_30_Ca_4_ and Mg_66_Zn_30_Ca_3_Sr_1_. In order to observe the variation of the electronic properties of Mg_66_Zn_30_Ca_4_ after the addition of Sr, the DFT calculation was employed to obtain the PDOS of s, p, and d orbitals of surface Mg, Zn, Ca, and Sr elements. The XRD profiles of both BMGs generated by the SABH method closely match the corresponding experimental XRD profiles, indicating that the SABH-predicted structures are the same as the experimental ones. The HA index analysis shows that the fractions of icosahedral-like local structures are about 72.20% and 72.73% for Mg_66_Zn_30_Ca_4_ and Mg_66_Zn_30_Ca_3_Sr_1_, respectively. These two BMG structures with the high icosahedral-like fractions are entirely amorphous, and the atomic arrangement of our configurations highly conforms to the experimental research. Uniaxial tensile MD simulation was conducted to obtain the stress-strain relationship as well as the related mechanical properties of Mg_66_Zn_30_Ca_4_ and Mg_66_Zn_30_Ca_3_Sr_1_. The predicted Young’s moduli of both BMGs are about 46.4 GPa, which is very close to the experimental values of 48.8 ± 0.2 and 49.1 ± 0.1 GPa for Mg_66_Zn_30_Ca_4_ and Mg_66_Zn_30_Ca_3_Sr_1_, respectively. The predicted strengths of Mg_66_Zn_30_Ca_4_ and Mg_66_Zn_30_Ca_3_Sr_1_ are about 850 MPa and 900 MPa, and these two values are slightly higher than that of the related experimental values, about 747 ± 22 and 848 ± 21 MPa for Mg_66_Zn_30_Ca_4_ and Mg_66_Zn_30_Ca_3_Sr_1_, respectively.

Regarding the thermal properties, the predicted melting temperature of Mg_66_Zn_30_Ca_3_Sr_1_ by the SD profile is about 620 K, which is very close to the experimental melting temperature of about 613 K. The self-diffusion coefficients of Mg, Zn, Ca, and Sr elements near their melting temperatures were obtained by the use of the Einstein equation, utilizing the MSD profile slope at the long-time limit. Through the use of these diffusion coefficients, the diffusion barriers for different elements can be determined. The diffusion barriers of Ca and Sr elements of Mg_66_Zn_30_Ca_3_Sr_1_ are relatively higher than the others. For the electronic properties predicted by the DFT calculation, the PDOS profiles of surface Mg, Zn, Ca, and Sr elements clearly exhibit the fact that the addition of Sr into Mg_66_Zn_30_Ca_4_ can effectively reduce the s and p orbital states of surface Mg and Zn elements near the Fermi level, which suppresses the electron transfer and increases the surface corrosion resistance of Mg_66_Zn_30_Ca_4_.

This study has provided the parametrization process to obtain excellent 2NN MEAM parameters for the Mg, Zn, Ca, and Sr system through the GCPSO method. With this 2NN MEAM potential, the SABH process can prepare the BMG structures with the same local atom arrangement as the experimental observation. The theoretically predicted structural, mechanical, and thermodynamic properties of Mg_66_Zn_30_Ca_4_ and Mg_66_Zn_30_Ca_3_Sr_1_ are all close to the experimental results. With these BMG structures, the DFT calculation was used to explore their electronic properties. Eventually, Through the numerical process in this study, the material properties of BMG with different compositions can be accurately predicted for the new BMG design.

## Supplementary information

Supplementary Information.

MgZnCaSr.meam.

MgZnCaSr_ cross_element.meam.
