# Supplementary material for: Effects of Strontium incorporation to Mg-Zn-Ca biodegradable bulk metallic glass investigated by molecular dynamics simulation and density functional theory calculation
Source: Sci Rep. 2020 Feb 13;10:2515. doi: 10.1038/s41598-020-58789-8 (PMC7018969; doi:10.1038/s41598-020-58789-8)
Supplement: Supplementary file 1 — Supplementary Information. [file 41598_2020_58789_MOESM1_ESM.pdf]

Effects of Strontium incorporation to Mg-Zn-Ca biodegradable bulk metallic glass investigated by molecular dynamics simulation and density functional theory calculation

Shih-Jye Sun[1], Shin-Pon Ju[2,3]*, Cheng-Chia Yang[2], Kai-Chi Chang[2] , and I-Jui Lee[2]

[1]Department of Applied Physics, National University of Kaohsiung, Kaohsiung 811, Taiwan

[2]Department of Mechanical and Electro-Mechanical Engineering, National Sun Yat-sen University, Kaohsiung 804, Taiwan

[3]Department of Medicinal and Applied Chemistry, Kaohsiung Medical University, Kaohsiung 807, Taiwan

*Corresponding author (jushin-pon@mail.nsysu.edu.tw)

The parametrization process

For $Mg_{66}Zn_{30}Ca_4$ and $Mg_{66}Zn_{30}Ca_3Sr_1$ BMGs, the interactions among four elements Mg, Zn, Ca, and Sr were modeled by the 2NN MEAM potential. Since the potential form and all parameters of 2NN MEAM have been thoroughly introduced in the original 2NN MEAM papers [1-6], we do not repeat that introduction here. The 2NN MEAM Mg and Ca parameter sets listed in Table S1 were used [7, 8]. For zinc, the available 2NN MEAM parameters by Lee *et al.* [9] cannot predict the correct a/c ratio of the HCP zinc unit cell. In Dickel's study [10], although their 2NN MEAM parameters can predict the experimental c/a ratio, the cross-element 2NN MEAM potential parameters fitted by particle swarm optimization (PSO) obtained an unreasonable shorter Zn-Zn bond length in the current BMG simulation. It could be attributed to the ranges of the $t^{(1)}$, $t^{(2)}$, and $t^{(3)}$ parameters are much larger than those of all available elements. Consequently, the hypothetical FCC zinc with some reference structures provided by the density functional theory (DFT) calculation was used to

obtain the 2NN MEAM parameters of zinc. For Sr, there are no available 2NN MEAM parameters in previous studies, so the 2NN MEAM Sr parameters were also fitted by PSO according to the reference data from the experiment or DFT calculation.

The guaranteed convergence particle swarm optimization (GCPSO) [11, 12] was used to fit the required 2NN MEAM parameters according to reference data prepared by the density functional theory (DFT) calculation or experimental data. The large-scale atomic/molecular massively parallel simulator (LAMMPS) [13] was used for obtaining the corresponding properties during the GCPSO process. The DMol3 package was used for all DFT calculations, and the generalized gradient approximation (GGA) with the parameterization of PBEsol was used [14]. For the DFT settings, all electron calculations used a double numeric plus polarization basis set DNP [15-18]. The energy tolerance in the self-consistent field calculations was 2.72×10^{-5} eV, and the energy, force and atomic displacement tolerances for the ionic step were 2.72×10^{-4} eV, 5.44×10^{-2} eV/Å and 5.00×10^{-3} Å, respectively. Table S2 lists the experimental and predicted binding energies and the lattice information as well as the related errors between them. Most errors are lower than 3%, indicating the current DFT setting can predict reliable material properties of Mg, Zn, Ca, and Sr at the same time, implying these DFT settings are also reliable for the Mg-Zn-Ca-Sr multiple element system.

For FCC zinc and FCC Sr, the binding energies, lattice constants, elastic constants, and the binding energy variation with the lattice constant change obtained by the DFT calculation were used as the GCPSO reference data. Because the binding energy and lattice constant are two major properties considered in the current study, the weightings of these two properties for the GCPSO target function are 100 times the ones for the elastic constants. Table S3 lists the 2NN MEAM parameters for Zn and Sr after the GCPSO fitting process, and the profiles for the binding energies versus the lattice constants can be seen in Fig. S1. The profiles from the 2NN MEAM potential closely

match those obtained from the DFT calculation. It indicates the 2NN MEAM parameters of Zn and Sr can basically reflect the material properties of Zn and Sr. In Table S4, the binding energies and lattice constants of Mg, Zn, Ca, and Sr are shown, and the binding energies and lattices from 2NN MEAM are very close to the experimental data and DFT calculation.

To obtain the cross-element 2NN MEAM parameters, the B1 structures, as shown in Fig. S2(a) (only ZnCa B1 was shown) for MgZn, MgCa, MgSr, ZnCa, ZnSr, and CaSr were used as the reference structures. Besides the B1 structures, the 3x3x2 BCC supercell with a total of 36 atoms (Fig. S2(b)) and the 2x2x2 FCC supercell with a total of 32 atoms (Fig. S2(c)) were used as the template structures. After randomly substituting atoms by Mg, Zn, Ca, and Sr atoms at an equal fraction of 25%, five BCC supercells and five FCC supercells were generated, which can be seen in Fig. S2(b) and Fig. S2(c) as examples. These structures, including six B1 structures, five FCC supercells, and five BCC supercells, were optimized entirely by the DFT calculation before the GCPSO fitting process. Reference data for the GCPSO fitting process were the binding energies, lattice constants, and binding energy variation with the lattice constant for the six B1 structures as well as the binding energies, shear stresses, and normal stresses for the five FCC supercells and five BCC supercells. The stabilities of the six B1 structures were not considered in the current fitting process, so the elastic constants of these hypothetical B1 structures were not considered in the GCPSO fitting. Figure S3 shows the variation of binding energy at different lattice constants for the ZnSr B1 structure. The results from the fitted 2NN MEAM parameters closely match those from the DFT calculation. Because the other five B1 structures show very similar results, these profiles are not shown here.

For convenience in presenting the fitting results for the FCC and BCC supercells, they are designated as Case 1 to Case 10 in Fig. S4. The first 5 cases are BCC, and the

last five are FCC. One can see the 2NN MEAM potential with the fitted parameters can predict very similar binding energy values as those from the DFT calculation because the errors are lower than 3%. Consequently, the 2NN MEAM potential with the GCPSO fitted Mg-Zn-Ca-Sr parameter sets and those for pure Mg, Zn, Ca, and Sr elements can be used to predict the material properties of the Mg-Zn-Ca-Sr BMG system. All 2NN MEAM parameters for the Mg-Zn-Ca-Sr system in the LAMMPS format are also provided with the supporting file (MgZnCaSr.meam for single element parameters and MgZnCaSr_ cross_element.meam for the cross-element parameters).

The stress calculation

During the tension process, the tensile stress at different strains was calculated by the sum of per-atom-stress (σi_{mn}) divided by the sum of atomic volume (V_i) as shown below:

$$\sigma_{mn} = \frac{\sum_i \sigma i_{mn}}{\sum_i V_i} \qquad (1)$$

where indexes of m and n represent the m plane and n-direction. The per-atom-stress of atom i (σi_{mn}) was directly calculated by LAMMPS [13].

The atomic volume of atom i (V_i) is defined by Eq. (2) [25]:

$$V_i = \frac{2\pi \sum r_{ij}{}^{-1}}{3 \sum r_{ij}{}^{-2}}$$

(2)

where r_{ij} is the interatomic distance between two atoms i and j within the first nearest neighbor distance, which can be obtained from the first minimal distance of the RDF first peak.

Table S1. 2NN MEAM parameter sets for pure Mg and Ca from previous studies [7, 8].

	E_c	r_e	B	A	$\beta^{(0)}$	$\beta^{(1)}$	$\beta^{(2)}$	$\beta^{(3)}$	$t^{(1)}$	$t^{(2)}$	$t^{(3)}$	C_{min}	C_{max}	d
Mg	1.550	3.200	0.369	0.52	2.30	1.00	3.00	1.00	9.00	-2.00	-9.50	0.49	2.80	0.00
Ca	1.840	3.946	0.183	0.40	4.00	2.20	2.00	2.20	1.70	-7.70	6.00	0.98	2.80	0.05

Table S2. The experimental and DFT predicted binding energies and lattice constants of Mg, Zn, Ca, and Sr unit cells.

**Element**	**Property**	**Exp**[19-24]	**DFT**	Error(%)
Mg (HCP)	Binding energy	-1.55	-1.72	-9.88
	Lattice constants a	3.20	3.16	-1.25
	Lattice constants c	5.21	5.17	-0.38
	c/a	1.62	1.64	1.23
Zn (HCP)	Binding energy	-1.35	-1.33	1.48
	Lattice constants a	2.66	2.72	2.26
	Lattice constants c	4.94	4.64	-6.07
	c/a	1.85	1.70	-8.10
Ca (FCC)	Binding energy	-1.84	-1.86	-1.00
	Lattice constants	5.58	5.51	-1.25
Sr (FCC)	Binding energy	-1.72	-1.71	0.58
	Lattice constants	6.08	6.09	0.16

Table S3. The 2NN MEAM parameter sets for Zinc and Strontium

Element	E_c	a_{lat}	α	A	$\beta^{(0)}$	$\beta^{(1)}$	$\beta^{(2)}$	$\beta^{(3)}$	$t^{(1)}$	$t^{(2)}$	$t^{(3)}$	C_{min}	C_{max}	Attrac	Repuls
Zn	1.31	3.93	6.76	1.20	6.38	0.00	4.72	9.91	10.00	5.055	10.00	1.2	2.5	0.00	0.00
Sr	1.72	6.083	4.59	0.249	3.89	6.49	6.90	0.0	-10.00	-10.00	10.00	0.9	2.9	0.00	0.00

Table S4. The predicted material properties by the 2NN MEAM and a comparison to the reference data from the experiment or DFT calculation. These material properties include the binding energy (eV/atom), lattice constant (Å), elastic constants (C_{11}, C_{12}, and C_{44}) (GPa) for pure Mg, Zn, Ca and Sr. The c/a ratio, C_{13}, and C_{33} of Mg are also shown.

Element	Property	Exp/DFT	2NN MEAM	Error(%)
Mg (HCP)	Binding energy	-1.55	-1.55	0.00
	Lattice constants a	3.18	3.209	0.94
	Lattice constants c	5.21	5.19	-0.38
	c/a	1.62	1.62	0.00
	C_{11}	63.50	62.90	-0.94
	C_{12}	25.90	26.10	0.77
	C_{13}	21.70	21.20	-2.30
	C_{33}	66.50	69.70	4.81
	C_{44}	18.40	17.10	-7.06
Zn (FCC)	Binding energy	-1.31	-1.31	0.00
	Lattice constants	3.93	3.93	0.00
	C_{11}	105.35	97.28	-7.66
	C_{12}	64.46	57.88	-10.21
	C_{44}	1.83	1.83	0.00
Ca (FCC)	Binding energy	-1.84	-1.84	0.00
	Lattice constants	5.59	5.58	-0.17
	C_{11}	22.80	23.48	2.98
	C_{12}	16.00	15.70	-1.87
	C_{44}	14.00	10.10	-27.85
Sr (FCC)	Binding energy	-1.72	-1.74	1.16
	Lattice constants	6.089	6.03	-1.03
	C_{11}	15.00	15.15	1.03
	C_{12}	10.10	9.97	-1.27
	C_{44}	9.90	9.87	-0.29

(a)

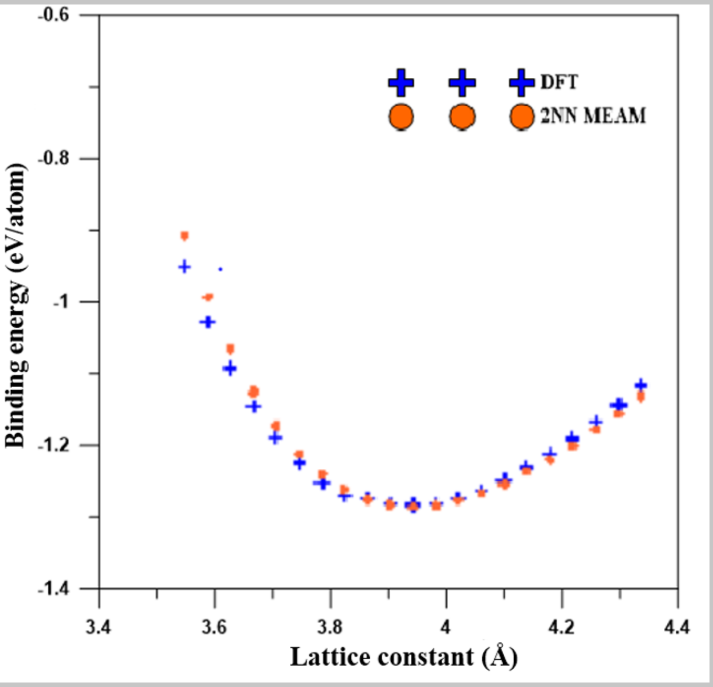

(b)

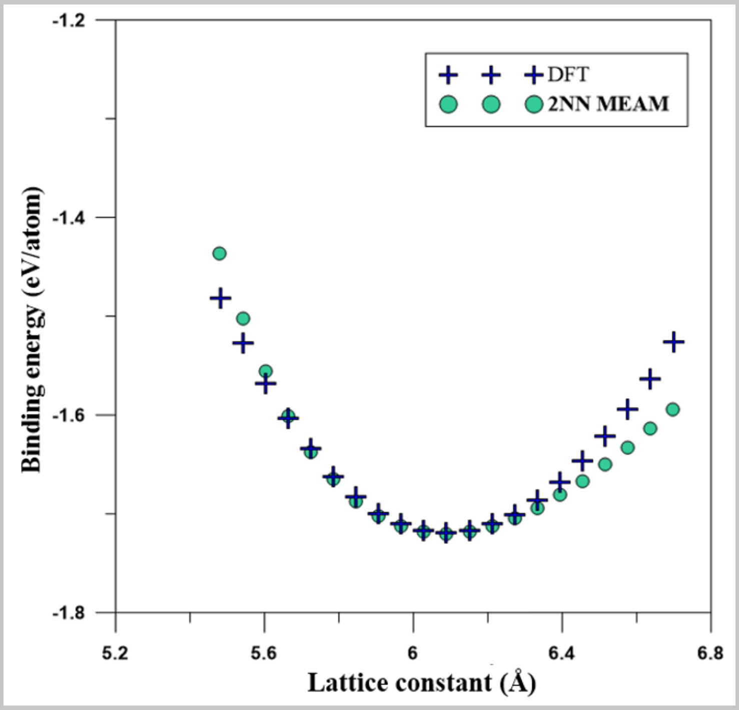

Fig. S1. The variations of binding energy versus lattice constant for (a) Zn and (b) Sr in FCC arrangements by the DFT calculation and the 2NN MEAM potential with the PSO fitted parameters.

(a)

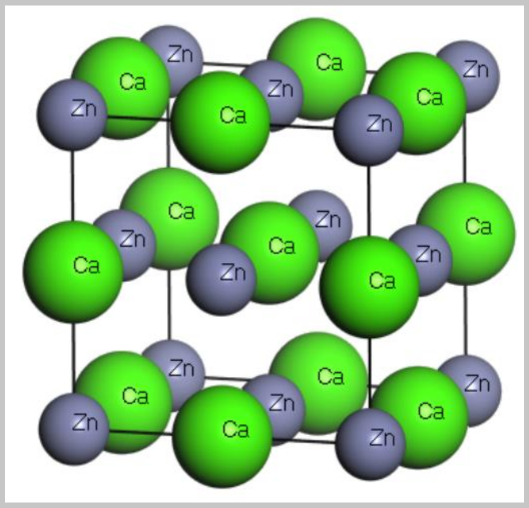

(b)

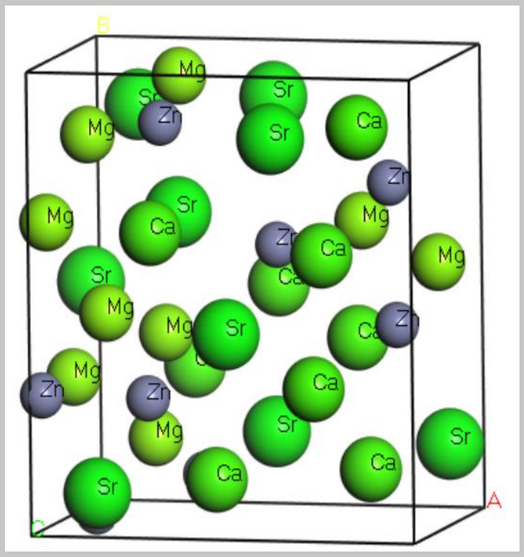

(c)

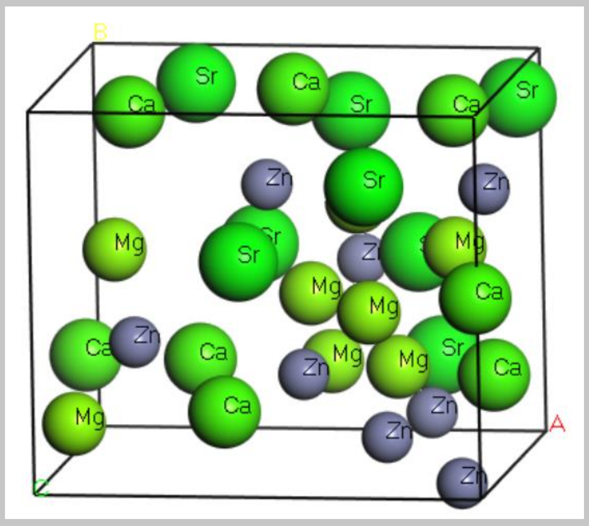

Fig. S2. The schematic diagrams of (a) a B1 unit cell for the binary system (b) an optimized BCC supercell for the Mg-Zn-Ca-Sr system, and (c) an optimized FCC supercell for the Mg-Zn-Ca-Sr system.

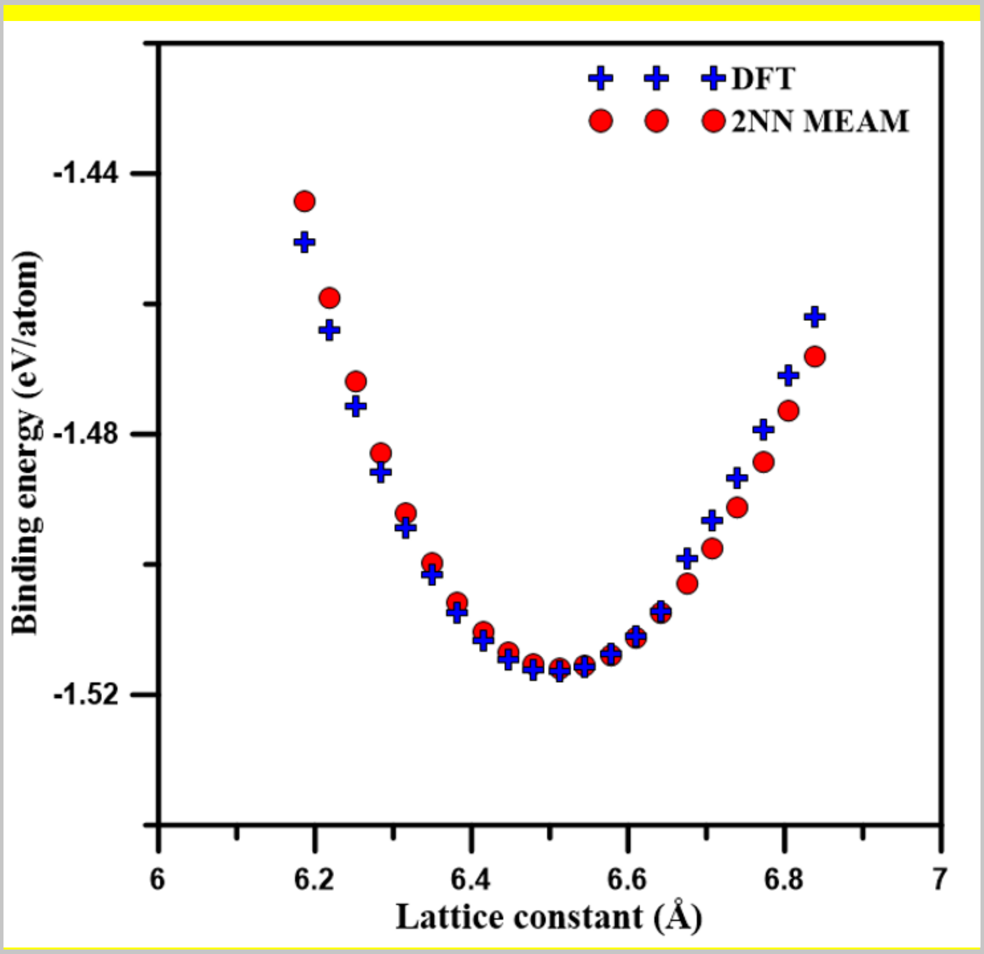

Fig. S3. The binding energies at different lattice constants for B1 ZnSr structure. The results from the DFT calculation and 2NN MEAM closely match. The binding energy profiles of the other binary B1 systems are also very similar and not shown here.

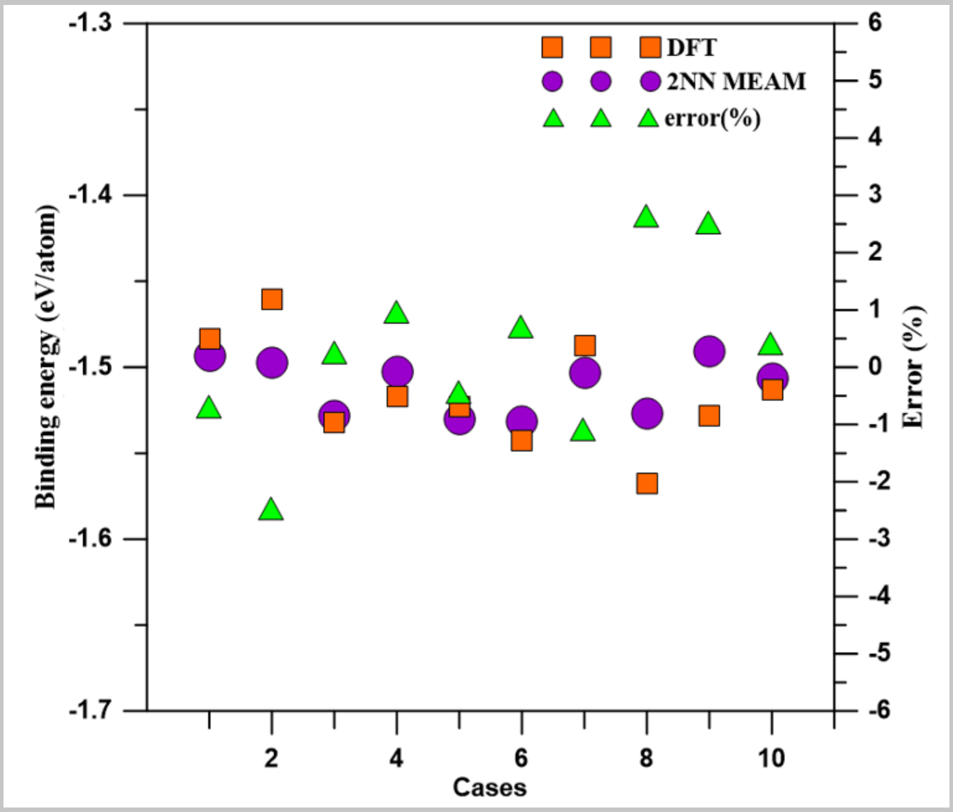

Fig. S4. The binding energy distributions of 5 BCC (the first 5 cases) and 5 FCC structures (the last five cases) by DFT calculation and 2NN MEAM with the fitted cross-element parameters. The absolute errors between the DFT and 2NN MEAM results for all cases are lower than 3%.

Reference

[1] M. Baskes, "Modified embedded-atom potentials for cubic materials and impurities," *Physical Review B,* vol. 46, no. 5, p. 2727, 1992.

[2] B.-J. Lee and M. Baskes, "Second nearest-neighbor modified embedded-atom-method potential," *Physical Review B,* vol. 62, no. 13, p. 8564, 2000.

[3] B.-J. Lee, M. Baskes, H. Kim, and Y. K. Cho, "Second nearest-neighbor modified embedded atom method potentials for bcc transition metals," *Physical Review B,* vol. 64, no. 18, p. 184102, 2001.

[4] B.-J. Lee, J.-H. Shim, and M. Baskes, "Semiempirical atomic potentials for the fcc metals Cu, Ag, Au, Ni, Pd, Pt, Al, and Pb based on first and second nearest-neighbor modified embedded atom method," *Physical Review B,* vol. 68, no. 14, p. 144112, 2003.

[5] B.-J. Lee, "A modified embedded-atom method interatomic potential for the Fe–C system," *Acta materialia,* vol. 54, no. 3, pp. 701-711, 2006.

[6] Y.-M. Kim and B.-J. Lee, "A semi-empirical interatomic potential for the Cu–Ti binary system," *Materials Science and Engineering: A,* vol. 449, pp. 733-736, 2007.

[7] Y.-M. Kim, N. J. Kim, and B.-J. J. C. Lee, "Atomistic modeling of pure Mg and Mg–Al systems," vol. 33, no. 4, pp. 650-657, 2009.

[8] K.-H. Kim, J. B. Jeon, and B.-J. J. C. Lee, "Modified embedded-atom method interatomic potentials for Mg–X (X= Y, Sn, Ca) binary systems," vol. 48, pp. 27-34, 2015.

[9] H.-S. Jang, K.-M. Kim, and B.-J. J. C. Lee, "Modified embedded-atom method interatomic potentials for pure Zn and Mg-Zn binary system," vol. 60, pp. 200-207, 2018.

[10] D. E. Dickel, M. I. Baskes, I. Aslam, C. D. J. M. Barrett, S. i. M. Science, and Engineering, "New interatomic potential for Mg–Al–Zn alloys with specific application to dilute Mg-based alloys," vol. 26, no. 4, p. 045010, 2018.

[11] R. Eberhart and J. Kennedy, "A new optimizer using particle swarm theory," in *Micro Machine and Human Science, 1995. MHS'95., Proceedings of the Sixth International Symposium on*, 1995, pp. 39-43: IEEE.

[12] F. van den Bergh and A. P. Engelbrecht, "A new locally convergent particle swarm optimiser," in *Systems, Man and Cybernetics, 2002 IEEE International Conference on*, 2002, vol. 3, p. 6 pp. Vol. 3: IEEE.

[13] S. Plimpton, P. Crozier, and A. Thompson, "LAMMPS-large-scale atomic/molecular massively parallel simulator," *Sandia National Laboratories,* vol. 18, pp. 43-43, 2007.

[14] J. P. Perdew *et al.*, "Restoring the density-gradient expansion for exchange in

solids and surfaces," vol. 100, no. 13, p. 136406, 2008.

[15] B. Delley, D. Ellis, A. Freeman, E. Baerends, and D. Post, "Binding energy and electronic structure of small copper particles," *Physical Review B,* vol. 27, no. 4, p. 2132, 1983.

[16] B. Delley, "An all-electron numerical method for solving the local density functional for polyatomic molecules," *The Journal of chemical physics,* vol. 92, no. 1, pp. 508-517, 1990.

[17] B. Delley, "Fast calculation of electrostatics in crystals and large molecules," *The Journal of Physical Chemistry,* vol. 100, no. 15, pp. 6107-6110, 1996.

[18] B. Delley, "From molecules to solids with the DMol 3 approach," *The Journal of chemical physics,* vol. 113, no. 18, pp. 7756-7764, 2000.

[19] C. Barrett and T. Massalski, "Structure of Metals: Crystallographic Methods, Principles, and Data pdf," 1952.

[20] M. Baskes and R. Johnson, "Modified embedded atom potentials for HCP metals," *Modelling and Simulation in Materials Science and Engineering,* vol. 2, no. 1, p. 147, 1994.

[21] G. Simmons and H. Wang, "Single crystal elastic constants and calculated aggregate properties," 1971.

[22] C. Kittel, P. McEuen, and P. McEuen, *Introduction to solid state physics*. Wiley New York, 1996.

[23] E. A. Brandes and G. Brook, *Smithells metals reference book*. Elsevier, 2013.

[24] M. Heiroth, U. Buchenau, H. Schober, and J. Evers, "Lattice dynamics of fcc and bcc calcium," *Physical Review B,* vol. 34, no. 10, p. 6681, 1986.

[25] D. Srolovitz, K. Maeda, V. Vitek, and T. Egami, "Structural defects in amorphous solids Statistical analysis of a computer model", Philos. Mag. A 44, 847, 1981.
